# 肺癌免疫治疗耐药机制及应对策略

**DOI:** 10.3779/j.issn.1009-3419.2021.101.02

**Published:** 2021-02-20

**Authors:** 雅凝 杨, 路 杨, 燕 王

**Affiliations:** 100021 北京，国家癌症中心/国家肿瘤临床医学研究中心/中国医学科学院北京协和医学院肿瘤医院内科 Department of Medical Oncology, National Cancer Center/National Clinical Research Center for Cancer/Cancer Hospital, Chinese Academy of Medical Sciences and Peking Union Medical College, Beijing 100021, China

**Keywords:** 免疫检查点抑制剂, 肺肿瘤, 耐药机制, 应对策略, Immune checkpoint blockade, Lung neoplasms, Resistance mechanism, Response strategy

## Abstract

近年来，免疫治疗在肺癌领域取得了令人瞩目的突破，为肺癌患者带来长久的生存获益。但是随着临床应用的逐渐广泛，在一线接受免疫检查点抑制剂治疗的患者中，约30%-50%表现为短暂获益或不获益，提示免疫耐药的存在。目前研究表明免疫耐药是一个综合的、动态的过程，其机制受到肿瘤细胞、免疫微环境及宿主本身等多方面的影响。随着免疫耐药机制的深入研究、免疫治疗新靶点的发现及免疫联合治疗的发展，免疫耐药后的治疗策略成为当今时代下的重要思考方向。本文将着重探讨以上问题，希望为筛选优势人群、扩大受益群体提供线索和思路。

## Abstract (en)

Inhibition of immune checkpoints is at the forefront of immunotherapy for lung cancer. However, a high percentage of lung cancer patients do not respond to these immunotherpy or their responses are transient, indicating the existence of immune resistance. Emerging evidence suggested that the interactions between cancer cells and immune system were continuous and dynamic. Here, we review how a range of cancer-cell-autonomous characteristics, tumor-microenvironment factors, and host-related influences account for heterogenous responses. Furthermore, with the identification of new targets of immunotherapy and development of immune-based combination therapy, we elucidate the methods might useful to overcome resistance.

## 1 背景

肺癌是中国及全世界发病率及死亡率第一的恶性肿瘤。随着免疫检查点抑制剂（简称“免疫治疗”）的问世，肺癌的治疗进入了免疫治疗时代。在新辅助治疗中，免疫检查点抑制剂联合化疗给可手术切除的早期非小细胞肺癌（non-small cell lung cancer, NSCLC）患者增加了更多治愈的机会。而在维持治疗中，Durvalumab明显延长了同步放化疗后的NSCLC的无病生存时间。接受免疫检查点抑制剂单药治疗的晚期NSCLC患者5年生存率从既往化疗时代的5%提高到15%。同时，程序性死亡配体1（programmed death-ligand 1, PD-L1）抗体联合化疗在晚期小细胞肺癌（small cell lung cancer, SCLC）的治疗史中也带来了里程碑式的突破。综上，随着对免疫治疗的不断探索，越来越多的肺癌患者得到长期生存获益。但是，在一线治疗接受免疫治疗的肺癌患者中，有7%-27%的患者出现原发耐药，这一比例在二线治疗中是20%-44%^[1]^。此外，根据KEYNOTE-001研究的结果，约有25%的患者出现继发耐药^[2]^。所以，明确耐药机制、筛选获益人群是当前精准医疗时代背景下亟待解决的问题。本文拟针对目前已知的免疫耐药机制及潜在的逆转免疫耐药的治疗策略进行综述。

## 2 耐药机制

### 2.1 肿瘤细胞的内部因素

肿瘤细胞的内部因素主要是指肿瘤细胞内特定基因和通路的表达或抑制，如驱动基因活化及抑癌基因失活、低肿瘤突变负荷及新抗原、肿瘤抗原递呈能力下降及肿瘤细胞表面PD-L1表达变化等均会导致免疫耐药的发生。

#### 2.1.1 驱动基因活化

肿瘤细胞的基因组或表观遗传的变化可介导免疫逃逸。肺癌有丰富的驱动基因，其中一些基因的改变如表皮生长因子受体（epidermal growth factor receptor, *EGFR*）、间变性淋巴瘤激酶（anaplastic lymphoma kinase, *ALK*）、鼠类肉瘤病毒癌基因（Kirsten rat sarcoma viral oncogene homolog, *KRAS*）等，均被研究证实与免疫逃逸过程相关。

*EGFR*基因突变是亚裔NSCLC中最为常见的突变类型，发生率为40.3%-64.5%^[3]^。根据以往临床试验结果，此类患者对免疫单药治疗反应不佳^[4-6]^。此类人群对免疫治疗效果不佳的原因有以下几种假说：①目前普遍被认可的原因是，该类患者的免疫微环境属于对免疫治疗效果欠佳的免疫沙漠型或者豁免型^[7, 8]^。EGFR通路激活后，配体双调蛋白（amphiregulin, AREG）可通过EGFR/糖原合成酶激酶-3β（glycogen synthase kinase-3β, GSK-3β）/叉头框蛋白3（forkhead box P3, FOXP3）轴促进调节性T（regulatory T, Treg）细胞的产生从而增强免疫抑制作用^[9]^。同时，EGFR信号通路还可产生抑制性细胞因子，诱导髓系来源的抑制性细胞（myeloid-derived suppressor cell, MDSC）和肿瘤相关巨噬细胞（tumor-associated macrophage, TAM）的增殖、抑制CD8^+^ T细胞的应答^[10]^。②EGFR通路激活时，其信号通路下游的信号转导及转录激活蛋白3（signal transducer and activator of the transcription 3, STAT3）被上调，继而导致主要组织相容性复合体（major histocompatibility complex, MHC）Ⅰ类分子的表达下降^[11]^。同时，STAT3也介导了血管内皮生长因子（vascular endothelial growth factor, VEGF）、白细胞介素-6（interleukin-6, IL-6）、白细胞介素-10（interleukin-10, IL-10）的表达，而这些因子进一步抑制了树突细胞（dendrite cells, DC）的分化和成熟。以上过程均会影响抗原的提呈，减少新抗原的产生。③尽管多数研究已证实EGFR激活后会上调PD-L1，但由于新抗原减少、免疫抑制细胞增多，故此类患者接受免疫单药治疗效果不理想^[10]^。

肺癌另一常见的基因突变则为*ALK*融合。*ALK*融合患者可通过PI3K-AKT及MEK-ERK通路减少新抗原的产生，增加免疫抑制细胞的数量，导致免疫单药治疗效果不佳^[12, 13]^。*KRAS*基因同样是肺癌发生的驱动基因，*KRAS*基因突变在白种人中的发病率较高，约为30%。*KRAS*突变可通过调节PD-L1 mRNA 3’UTR区的稳定性，上调PD-L1表达促进免疫逃逸^[14]^。但是对于免疫检查点抑制剂，KRAS却表现出了良好的反应性。在一项单中心回顾性研究中，纳入了25例*KRAS*突变和47例野生型，予以Nivolumab±抗CTLA-4抗体，两组的中位总生存时间（overall survival, OS）分别为18.1个月 *vs* 8.1个月（HR=0.48, *P*=0.04）^[15]^。究其原因，可能是由于*KRAS*突变上调新抗原的表达并改变了细胞周期调控、DNA复制及DNA修复等一系列基因，但是没有明显的活化免疫抑制细胞作用^[16]^。

#### 2.1.2 抑癌基因失活

抑癌基因，如*PTEN*、*STK11*基因的失活，同样也可以导致肺癌患者使用免疫治疗效果不佳。在耐药人群中，抑癌基因*PTEN*突变明显增加^[17]^。PTEN是抑制PI3K通路活性的脂质磷酸酯酶，目前研究^[18]^表明，PTEN表达缺失是多个瘤种中活化PI3K通路最常见的方式。而PI3K通路通过调节包括肿瘤增殖、存活在内的一些关键的细胞加工处理过程，在肿瘤生长中起着不可或缺的作用。另有研究^[19]^发现PTEN缺失率高的患者肿瘤浸润性淋巴细胞（tumor-infiltrating lymphocyte, TIL）表达减少。同时，PTEN可以上调VEGF的表达，进一步地阻止淋巴细胞浸润而介导肿瘤的免疫逃逸。*STK11*/*LKB1*基因也是抑癌基因，在NSCLC中约有8%-39%的患者存在*STK11*基因突变，其功能主要为负向调节雷帕霉素信号途径。*STK11*基因突变常伴随浸润性细胞毒性CD8^+^ T淋巴细胞的减少，导致冷肿瘤的免疫微环境^[20]^。Skoulidis等^[21]^的研究中共纳入了165例接受过PD-1/PD-L1抑制剂治疗的*KRAS*突变肺腺癌患者，结果表明携带*KRAS*突变及*STK11*/*LKB1*改变的患者接受免疫治疗效果不佳，在同基因小鼠*KRAS*突变模型中，*STK11*基因缺失促进PD-1抑制剂单药治疗的原发耐药。*SMARCA4*基因在NSCLC中的突变率约为10%，它负责编码SWI/SNF染色质重构复合物的催化单位。在2020年美国肿瘤学年会中，一项研究^[22]^共纳入了2, 690例NSCLC患者，其中211例患者携带*SMARCA4*突变。研究者对于共突变进行了进一步的分析，其中，*KRAS*和*SMARCA4*共突变是最为多见的，在接受免疫治疗的*KRAS*突变的NSCLC患者中，并发*SMARCA4*突变出现了客观缓解率（objective response rate, ORR）显著降低（23.5% *vs* 0.0%, *P*=0.02）、无进展生存期（progression-free survival, PFS）显著缩短(4.1个月 *vs* 1.7个月，*P* < 0.001）以及中位OS明显缩短（14.9个月 *vs* 4.0个月，*P* < 0.001）。

综上，抑癌基因及促癌基因的突变可以导致肿瘤新抗原的变化，也可以影响免疫微环境的组成成分比例，故而体现为免疫治疗的疗效差异，但是由于免疫微环境受多种因素影响，所以体系基因突变的改变可以给临床治疗提供一些提示，但目前仍需解读其在免疫治疗的疗效及耐药的预测价值。

#### 2.1.3 肿瘤突变负荷（tumor mutation burden, TMB）及新抗原降低

TMB即所评估基因的外显子编码区每兆碱基中发生替换、插入、缺失突变的数量。Yarchoan等^[23]^通过对27个瘤种的TMB进行分析，研究发现TMB高的瘤种，免疫抑制剂单药治疗的ORR也更为理想。NSCLC、黑色素瘤等具有更高的突变负荷，免疫原性也相对更高，对免疫检查点抑制剂治疗更为敏感。相反地，如胰腺癌、前列腺癌、甲状腺等瘤种突变负荷较低而表现出免疫低应答^[23-25]^。肿瘤新抗原则是指在正常组织中不表达而仅在肿瘤组织中表达的抗原，通常具有强免疫原性。多项研究^[24, 26]^表明，TMB增高、DNA错配修复（DNA mismatch repair, dMMR）基因缺失、高基因组微卫星不稳定性（microsatellite instability, MSI）均可增加肿瘤抗原表达，提高免疫疗效。2017年Anagnostou等^[27]^发现在接受Pembrolizumab治疗的NSCLC患者人群中，持续临床获益的患者新抗原负荷要明显高于非持续获益的患者。而对治疗过程中出现耐药的患者研究发现，治疗前至耐药过程中将会丢失7个-18个能产生有效应答的突变相关新抗原，取而代之的则是更复杂的新生突变，新生突变参与编码肿瘤抗原的比例下降（8% vs 19%）且改变T细胞受体（T cell receptor, TCR）克隆性，从而导致获得性耐药的产生。

#### 2.1.4 肿瘤抗原递呈能力下降

主要组织相容性复合体（major histocompatibility complex, MHC）是参与免疫应答的关键分子，参与加工、处理和提呈抗原，从而激活T细胞并介导免疫应答。β2微球蛋白（β2-microglobulin, β2-GM）是组成MHC Ⅰ类分子重链的重要蛋白之一，主要负责将MHC Ⅰ类分子折叠并转运到细胞表面^[28]^。研究^[29]^证实在NSCLC中，*B2M*基因突变会导致细胞表面MHC Ⅰ类分子缺失，进而促进CD8^+^ T细胞的识别障碍，诱导免疫耐药。此外有研究^[30]^发现在肿瘤细胞和浸润的淋巴细胞中MHC Ⅱ类分子也存在表达缺陷，因此不能有效地激活T辅助细胞。较低的抗原递呈能力影响了肺癌的免疫原性，而弱免疫原性的肿瘤能逃脱免疫系统的监视，更易发生免疫逃逸，促使了部分肺癌免疫治疗耐药。

#### 2.1.5 PD-L1表达变化

肿瘤细胞高表达PD-L1同样也可以介导免疫逃逸，与T细胞的PD-1受体结合时则可传递负性调控信号，诱导T细胞凋亡或使其丧失功能，从而逃避免疫系统的攻击。在获得性免疫耐药患者中，研究发现TIL分泌的γ-干扰素（interferon-γ, IFN-γ）和肿瘤抗原特异性T细胞均可在肿瘤抗原识别后介导PD-L1表达的上调。此外，肿瘤尚有多种途径可上调PD-L1表达从而起到免疫逃逸的作用，如目前已知的*PTEN*缺失^[18]^、*PI3K*或*AKT*突变^[31]^等都可使肿瘤细胞组成性表达PD-L1从而产生耐药。但目前如KEYNOTE-024、KEYNOTE-208的临床试验证实PD-L1高表达可使肿瘤细胞对免疫治疗更敏感^[32, 33]^，中国临床肿瘤学会（Chinese society of Clinical Oncology, CSCO）指南和美国国家综合癌症网络（National Comprehensive Cancer Network, NCCN）指南也因此说明PD-L1表达水平可作为使用免疫治疗的预测指标之一。这些研究提示我们，PD-L1的功能可能是多样的，而非绝对地“促进”或“抑制”。近期的一项研究^[34]^证实，去乙酰化依赖的PD-L1核易位促进免疫逃逸，还能够通过上调肿瘤细胞的PD-L2和VISTA等免疫检查点基因，从而促进肿瘤细胞对PD-1/PD-L1阻断疗法的耐药性产生。此外，核PD-L1还能够强烈诱导免疫应答相关基因，包括Ⅰ型和Ⅱ型IFN信号通路，核因子κB（nuclear factor kappa-B, NF-κB）信号通路和抗原递呈通路。这些结果均表明，PD-L1具有双重作用，而究竟是什么机制决定了PD-L1激活耐药或应答通路，仍需进一步的阐述。

#### 2.1.6 表观遗传学调控

表观遗传学是在基因的核苷酸序列不发生改变的情况下所致基因表达的变化，包括DNA甲基化、组蛋白修饰、核小体重构和非编码RNA表达的改变。表观遗传学的改变可影响免疫检查点的表达，破坏抗原递呈过程，抑制T细胞向肿瘤微环境的迁移和T细胞的活化，转录因子的螺旋化也会影响记忆T细胞的产生^[35]^。此外，miRNA也参与了上述过程，目前的研究^[35]^表明miR-214、miR-126、miR-568均可促进Treg细胞的生成及其功能，从而导致T细胞耗竭。

### 2.2 肿瘤细胞的外部因素

肿瘤细胞的外部因素主要是指微环境，肿瘤微环境是肿瘤细胞赖以生存和发展的内环境，也是免疫细胞杀灭肿瘤细胞的“主战场”。微环境是免疫正负功能相融的场所，免疫学家过去试图通过正向提高免疫能力，如用细胞因子、过继免疫治疗等治疗增强免疫治疗的疗效，结果却不尽人意。越来越多的研究者们将目光投向肿瘤微环境中的“油门”（如免疫效应细胞）和“刹车”（负向调控因子如Treg细胞、MDSC等）的微妙平衡，通过微环境中分子的调节来增强免疫治疗的作用。

#### 2.2.1 免疫效应细胞

免疫效应细胞较低的浸润水平及功能耗竭导致了免疫抑制从而介导了免疫逃逸。2017年*Nature*上的综述提出了一种新的免疫肿瘤分类，根据CD8^+^ T细胞的分布频率将免疫表型分为免疫炎症型、免疫豁免型及免疫沙漠型三种。在一项Durvalumab联合奥拉帕利联合治疗SCLC的Ⅱ期临床试验中，共纳入了14例可评估的患者，其中9例表现为免疫豁免型，3例表现为免疫炎症型，2例表现为炎症沙漠型^[36]^，而免疫治疗药物更倾向于在CD8^+^ T细胞浸润的肿瘤微环境中发挥作用。Guo等^[37]^分析了14例NSCLC患者外周血、癌组织及癌旁组织的12, 346个T细胞的单细胞测序，研究发现浸润T细胞主要包括3个亚群，除了耗竭细胞，另2个亚群表现为低耗竭状态，并与耗竭细胞可能存在转化关系，被定义为“耗竭前细胞”。相比于耗竭细胞，“耗竭前”细胞与肺腺癌患者的预后显著相关。NK细胞不受MHC限制、不依赖于抗体，可直接释放如穿孔素、TNF等杀伤介质发挥其免疫功能。NK细胞功能异常时，可通过如活化受体表达或过表达抑制性受体、生成细胞因子受损等途径实现免疫逃逸。Trefny等^[38]^分析了35例患者接受纳武利尤单抗治疗的NSCLC患者，发现NK细胞表达的免疫球蛋白样受体基因*KIR3DS1*与疗效呈相关性，基因突变可导致对免疫治疗的原发耐药。此外，今年的几项研究均对B细胞及三级淋巴结构（tertiary lymphoid structures, TLS）免疫应答关系进行了分析。三级淋巴结构是指免疫相关淋巴组织，包括黏膜相关淋巴组织、皮肤相关淋巴组织等。研究表明与免疫无应答的患者相比，免疫治疗有效的患者在治疗前B细胞相关的基因表达水平明显升高。与免疫治疗无效的患者比，免疫治疗有效的患者在组织中CD20阳性的B细胞和TLS密度以及TLS和肿瘤面积比都明显偏高^[39-41]^。以上研究均表明免疫效应细胞功能对于免疫治疗疗效起着关键调节作用。

#### 2.2.2 Treg细胞

Treg细胞是一类具有免疫抑制作用的T细胞群，可以抑制CD4^+^或CD8^+^ T细胞的活化、增殖，并能抑制初始T细胞和记忆性T细胞的功能。Treg细胞对于维持自身免疫平衡有着不可或缺的作用，可有效地削弱自身抗原引起的免疫，但也被许多可以表达自身抗原的肿瘤所“利用”从而逃避免疫侦查和杀伤^[42]^。Treg细胞介导免疫逃逸的机制主要包括：①以颗粒酶和穿孔素依赖方式介导效应细胞溶解; ②分泌可溶性或膜结合的抑制性细胞因子如跨膜型转化生长因子（transforming growth factor, TGF）-β、IL-10、IL-35，下调靶细胞表达IL-2Rα，抑制靶细胞增殖; ③下调抗原提呈细胞（antigen-presenting cells, APC）表达CD80和CD86等共刺激分子，干扰T细胞活化; ④阻断效应细胞的代谢^[43, 44]^。在肺癌、乳腺癌、胰腺癌等均发现升高的Treg含量^[45]^，而手术切除肿瘤后的Treg水平则显著下降^[46]^。动物实验^[47]^证明CD25^+^ Treg细胞的耗竭有效地提高了小鼠的抗肿瘤免疫反应。目前认为，消除Treg细胞应当作为治疗中的必要组成部分，已知的非特异性干扰Treg细胞功能的几种化学治疗药物有环磷酰胺、吉西他滨、甲氨蝶呤、沙利度胺等^[48]^。Tregs的靶向药物则是作用于Treg标记物如CD25、CTLA-4而选择性地消除肿瘤相关的Treg细胞，如我们所熟知的CTLA-4抗体Ipilimumab、Tremelimumab。除此以外，在转移性乳腺癌中进行Ⅰ期临床试验的抗CD25抗体Daclizumab^[49]^、抗OX40（CD134）单克隆抗体^[50]^、趋化因子受体8（chemokine receptor 8）^[51]^等均在试验中证实可消除肿瘤微环境中的Treg细胞，为癌症患者带来新的治疗希望。

#### 2.2.3 骨髓来源的抑制性细胞（myeloid-derived suppressor cells, MDSCs）

MDSCs是一类具有免疫抑制功能的细胞群，可以通过多种途径介导免疫逃逸和免疫耐受^[52]^。MDSCs除分泌TGF-β和IL-10等直接抑制T效应细胞的分子外，还具有诱导其他免疫抑制细胞的产生，多个细胞试验^[53, 54]^证明在肝癌、黑色素瘤等瘤种中MDSCs通过分泌IFN-γ和IL-10介导产生FOXP3^+^ Treg细胞。除此以外，MDSCs还可通过阻断淋巴细胞归巢、调节腺苷代谢所需酶等多种途径发挥免疫抑制作用。Kim等^[55]^发现对ICB耐药的荷瘤小鼠的MDSC水平较非荷瘤小鼠增加了5倍-7倍，同时发现MDSCs高表达PI3Kγ，PI3Kγ可促进炎性介质及免疫抑制因子的产生。应用PI3K抑制剂联合PD-1/CTLA-4抑制剂后，荷瘤小鼠MDSCs降至与对照组大致持平并可逆转耐药现象。De Henau等^[56]^再次验证PI3Kγ抑制剂（如PI-549）可以重塑免疫微环境，延缓肿瘤生长。在免疫耐药的肺癌模型中发现MDSC上有高表达的吲哚胺2, 3双氧化酶（indoleamine 2, 3-dioxygenase, IDO），既往研究^[57]^表明IDO的表达水平与临床分期呈正相关，应用IDO抑制剂INCB023843下调IDO的表达及MDSCs的比例，并能增加CD8^+^ T细胞的浸润，从而起到再活化T细胞抗肿瘤应答的作用^[58]^。这些研究则显示了抑制MDSCs的治疗联合免疫检查点抑制剂治疗的潜在价值。

#### 2.2.4 TAMs

在肿瘤进展的过程中，循环中的单核细胞和巨噬细胞被招募到肿瘤中，改变肿瘤微环境、加速肿瘤的进展。巨噬细胞可以根据肿瘤和基质细胞产生的信号变换功能表型。基于巨噬细胞的功能，我们将其分为2类：M1型巨噬细胞与炎症反应、清除病原体和抗肿瘤免疫有关; 而M2型巨噬细胞则恰恰相反，其影响抗炎反应、伤口愈合并具有促肿瘤特性。TAMs则与M2型巨噬细胞极为相似^[59]^。包括乳腺癌^[60]^、卵巢癌^[61]^和NSCLC^[62]^在内的多个瘤种的基础研究显示巨噬细胞的聚集与CCL2水平呈正相关。除CCL2外，其他趋化因子如CCL3、CCL4、CCL5，细胞因子如集落刺激因子（colony-stimulating factor-1, CSF-1）等也都参与了巨噬细胞的募集过程^[63, 64]^。TAMs通过减弱抗原提呈能力、释放如IL-10、TGF-β等免疫抑制因子来抑制T细胞的功能。目前克服TAMs所致免疫耐药的策略主要包括以下几种：抑制巨噬细胞的募集; 将M2型巨噬细胞转化为M1型和消除TAMs。现有研究证实通过趋化剂的调控抑制巨噬细胞的募集是可行有效的手段，例如，用Bindarit抑制CCL2^[65]^; 单克隆特异性抗体阻断VEGFR2减少巨噬细胞浸润和肿瘤生长^[66]^; CSF-1R阻断也可减少TAMs的数量、活化TME中的T细胞^[67]^。以上临床前试验均为逆转TAMs所致的耐药性提供了可靠依据。

#### 2.2.5 肿瘤微环境的代谢变化

肿瘤环境中的代谢改变能够通过产生免疫抑制代谢物抑制免疫细胞浸润等降低免疫效应^[68]^。如部分肿瘤以谷氨酰胺为供能物质，代谢分解的氨可激活临近免疫细胞的自噬。精氨酸代谢在T细胞活化和调节免疫反应中也有重要作用，TME中表达精氨酸酶1（arginase 1, ARG1）的免疫调节细胞（如M2型巨噬细胞、Treg细胞等）通过降解精氨酸限制T细胞对精氨酸的利用，从而抑制T细胞的功能。因此，在TME中抑制精氨酸酶可增强免疫检查点抑制剂的疗效，如ARG1抑制剂INCB001158与Nivolumab联合使用的临床试验正在进行中。在肿瘤中，脂肪酸合成速度往往会加快以产生细胞膜磷脂和信号分子。靶向这些代谢途径是增强抗肿瘤免疫的一种途径，利用胆固醇酯化酶ACAT1抑制剂Avasimibe能够改善效应T细胞的功能。近年来越来越多的研究聚焦在肿瘤微环境的代谢过程，为克服免疫耐药提供了新的思路。

综上所述，肿瘤微环境是免疫耐药机制中的重要组成部分，但是外部环境的异常大多是在内部环境改变的影响下发生的次生危机。如*EGFR*突变导致免疫微环境沙漠型或豁免型; CCL2增加导致M2型巨噬细胞的募集等。现有的治疗方式主要致力于通过联合其他药物增加新抗原或者抑制信号通路进而改变免疫微环境。例如，在IMpower150研究中，免疫联合化疗及抗血管治疗有效地改善EGFR阳性患者的免疫治疗疗效。因此，在免疫单药耐药患者中，通过联合治疗方式重启患者的免疫治疗也是未来的治疗方向选择之一。

### 2.3 宿主相关因素

除外上述的肿瘤内部因素和免疫微环境可能增加免疫抑制作用导致耐药外，其他方面如宿主相关因素也会影响免疫抑制剂的疗效。宿主相关因素主要包括一般状况（如年龄、体重）、吸烟史、肠道菌群分布、既往基础疾病以及抗生素或激素药物使用情况等。

随着年龄的增长，免疫系统功能随之退化，如APC及淋巴细胞的数量和功能均较前减退^[69]^。在一项纳入了538例患者的分析^[70]^中发现，患有黑色素瘤的老年患者对免疫治疗的反应比年轻人更好。另外，在小鼠模型中，年轻小鼠的肿瘤中有较多的Treg细胞和较少的细胞毒性T细胞，所以年轻小鼠接受免疫治疗的效果更佳。性别方面，有一项*meta*分析^[71]^发现男性患者接受免疫治疗的效果更佳。该研究共纳入了11, 351例晚期或发生转移的癌症患者，其中NSCLC患者3, 482例（31%），与对照组治疗相比，用免疫检查点抑制剂治疗的男性患者的合并总体生存HR为0.72，而女性为0.86，疗效存在显著差异（*P*=0.001, 9）。考虑这项研究纳入偏倚以及评价指标，其实际临床意义较为有限。除此以外，有研究提示体重也会影响免疫治疗的疗效。2019年*JAMA Oncology*上报道了Atezolizumab治疗晚期NSCLC患者的生存率与体重指数（body mass index, BMI）之间的关系，研究^[72]^对OAK、POPLAR、BIRCH和FIR四项研究进行了汇总分析，分析发现，体型肥胖与接受PD-L1抑制剂治疗患者的OS显著提高有关，且肥胖患者的死亡风险降低了64%（HR=0.36, 95%CI: 0.21-0.62）。对于肺癌患者而言，吸烟也是至关重要的因素。吸烟者基因突变的平均频率比不吸烟者高10倍以上，因此对免疫检查点抑制剂反应更佳^[73]^。近期的研究^[74]^报道香烟中的苯并芘可以诱导PD-L1水平升高，进一步解释了吸烟患者接受免疫治疗效果更佳的原因。除此以外，近年来越来越多的研究^[75]^表明，肠道菌群对于免疫治疗的疗效有着不可小觑的影响。在抗CTLA-4抗体作用的过程中，脆弱拟杆菌可以促进IL-12依赖性的Th1细胞发挥抗肿瘤作用。在小鼠模型中喂养双歧杆菌可以帮助恢复CTL应答、活化瘤内DC对抗原的处理、提呈过程，同时可诱导微环境中TIL的增加，从而增强抗肿瘤反应^[76]^。

## 3 克服耐药治疗策略

免疫系统不是简单地协同或者拮抗，而是有机地整合。肿瘤的耐药机制不是简单的肿瘤对抗免疫系统的过程，而是肿瘤细胞、免疫微环境、宿主因素等相互作用的结果。因此，免疫耐药后的治疗策略也应综合评估患者的肿瘤情况及免疫状况，深层次分析耐药原因后予以精准个体化的治疗方案。针对免疫耐药后的治疗一要对因、二需对症，促进免疫激活、启动T细胞应答时，还需避免T细胞耗竭、阻断TME中的免疫抑制因子作用。

### 3.1 免疫联合治疗

ICBs耐药后尚无标准治疗方案，目前最重要且最有效的措施是免疫联合治疗，旨在将无或低免疫应答的“冷”肿瘤变成反应性良好的“热”肿瘤。多种联合治疗策略如免疫联合靶向治疗、化疗、放疗等可从不同免疫反应阶段调整免疫应答、纠正耐药原因。

#### 3.1.1 免疫联合免疫治疗

抗肿瘤免疫反应涉及抗原识别、呈递，免疫细胞的激活。目前在肿瘤免疫治疗领域中，研究较多的免疫检查点抑制剂主要针对于PD-1、PD-L1、淋巴细胞激活基因3（lymphocyte activating gene 3, LAG-3）、T细胞免疫球蛋白黏蛋白-3（T cell immunoglobulin domain and mucin domain-3, TIM-3）及T细胞免疫球蛋白和ITIM结构域蛋白（T cell immunoglobulin and ITIM domain protein, TIGIT）这5个检查点。基于不同的原理，上述免疫检查点抑制剂联合应用可有增效作用。最为常见的免疫联合免疫治疗策略为PD-1抑制剂联合抗CTLA-4抗体。由于两者作用机制不同，联合使用可起到协同作用，既能通过拮抗CTLA-4在免疫反应早期诱导大量T细胞的产生，又可阻断PD-1与PD-L1/L2的结合恢复T细胞对肿瘤细胞的杀伤功能，还可减少T细胞的耗竭。CheckMate 227及CheckMate 032研究均证实免疫双药组的疗效优于免疫单药治疗^[77, 78]^。LAG-3表达于活化的CD4^+^、CD8^+^ T细胞、NK细胞及Treg细胞等表面，可结合其配体纤维蛋白介素-1（fibrinogen-like protein-1, FGL1）后抑制T细胞功能。LAG-3抗体MK-4280联合Pembrolizumab治疗晚期NSCLC的试验（KEYNOTE-495/NCT03516981）共招募了33例其他治疗失败的实体瘤患者，单药治疗组的DCR为17%，联合治疗组的DCR则达到了40%。TIGIT同样是一种免疫检查点蛋白，并且在多种免疫细胞中表达。TIGIT是CD226共刺激受体的特异性负调节子，TIGIT和PD-1/PD-L1通路均在免疫抑制中发挥重要作用。此外，TIGIT表达与T淋巴细胞耗竭有非常重要的联系，因此，阻断TIGIT对逆转PD-1/PD-L1抑制剂的耐药具有一定的作用。2020年ASCO会议上披露了CITYSCAPE研究的初步结果，这项小型2期随机双盲试验共入组了PD-L1阳性、未经化疗治疗的NSCLC患者135例，1:1随机分配到TIGIT抗体Tiragolumab联合Atezolizumab（TA组）和安慰剂联合Atezolizumab（PA组），结果显示在意向性治疗人群中，与PA组相比，TA组的ORR有所改善（31.3% *vs* 16.2%），中位PFS获得提升（5.4个月 *vs* 3.6个月），疾病进展风险下降43%，联用效果更佳^[79]^。此外，Relatimab联合Nivolumab治疗实体瘤的Ⅱ期临床试验（NCT03607890）及IMP321联合Pembrolizumab治疗晚期NSCLC患者的Ⅱ期临床试验（NCT03625323）均在开展中。Pembrolizumab联合TIGIT抗体治疗转移性实体瘤患者的Ⅰ期临床试验（NCT02964013）及TIM-3抑制剂联合PD-L1抗体治疗晚期复发/难治性实体瘤的Ⅰ期试验（NCT03099109）正在进行之中，期待有所突破。

#### 3.1.2 免疫联合化疗

免疫联合化疗不仅可以增加树突细胞的交叉呈递抗原作用^[80]^、也可以弱化肿瘤微环境的免疫抑制成分^[81]^，如Treg细胞、MDSCs、免疫抑制细胞因子等，继而达到增加毒性淋巴细胞与调节性T细胞的比例等作用^[82]^。KEYNOTE-189研究^[83]^显示，晚期NSCLC患者一线使用Pembrolizumab联合化疗比单独化疗效果更好，两组的OS分别为22.0个月和10.7个月，PFS为9.0个月和4.9个月，联合组的PFS及OS均得到了大幅延长。此外，CheckMate012、KEYNOTE-021、IMpower130、IMpower131研究等均表明，在不同的免疫抑制剂中，免疫联合化疗相较化疗均有更大的生存获益。基于目前的研究，美国食品药品监督管理局（Food and Drug Administration, FDA）先后批准了Nivolumab联合标准一线治疗EGFR/ALK野生型的NSCLC以及Pembrolizumab联合培美曲塞铂类化疗用于EGFR/ALK野生型PD-L1阳性非鳞NSCLC治疗。而在SCLC方面，一项双盲、随机、安慰剂对照的Ⅲ期研究IMpower133中，相比单纯化疗，Atezolizumab联合化疗一线治疗广泛期SCLC更是近二十年来第一个取得OS获益的研究^[84]^，在SCLC治疗中具有突破性的里程碑意义。

#### 3.1.3 免疫联合靶向治疗

靶向治疗可以增加肿瘤抗原、在T细胞启动、运输、瘤内浸润等多个步骤与免疫治疗起到协同增效作用。抗血管生成药物可以促进血管正常化，帮助免疫效应细胞浸润到瘤内，降低免疫抑制性细胞活性，重塑肿瘤微环境。

IMpower150研究评估了Atezolizumab联合贝伐珠单抗及化疗一线治疗非鳞NSCLC患者，包括*EGFR*及*ALK*突变人群，结果证实四药联合方案可以给晚期NSCLC患者带来明显的生存获益^[85]^。COSMIC-021研究的队列7纳入了30例驱动基因阴性既往接受过免疫检查点抑制剂治疗的晚期NSCLC患者，使用Atezolizumab联合卡博替尼治疗，结果显示ORR为23%，DCR为83%，中位持续缓解时间为5.6个月^[86]^。一项Ⅰ期随机临床研究则是探索了Nivolumab联合厄洛替尼治疗晚期*EGFR*突变阳性NSCLC患者的效果，共纳入了20例既往EGFR-TKI治疗后耐药的晚期NSCLC及1例未经EGFR-TKI治疗的患者，结果显示患者的中位PFS是5.1个月^[87]^。此外，还有一些新型靶向药物与免疫治疗的联合应用也取得了不俗的成绩。Nivolumab联合小分子多靶点抑制剂Sitravatinib治疗对免疫治疗耐药的晚期非鳞NSCLC患者，ORR达到了32.14%，中位PFS为6.8月，中位OS为15.1个月^[88]^。Pembrolizumab联合JAK1抑制剂Itacitinib治疗PD-L1表达 > 50%的NSCLC患者，总体ORR达到66.7%。随着精准化治疗的发展，免疫联合靶向治疗有着非常广阔的发展空间。

#### 3.1.4 免疫联合放疗

放疗通过对DNA的损伤导致肿瘤细胞死亡，现有研究^[89]^表明，放疗对免疫系统也有影响：①放疗可促进肿瘤新抗原的产生，并可活化抗原递呈的DC; ②同时可以改变肿瘤微环境，尤其是对血管的作用，可有效促进淋巴细胞的浸润; ③可以调节免疫检查点配体的表达。此外，局部放疗远隔效应，即可以消灭/缩小远端转移部位（非放射部位）的肿瘤。因此免疫联合放疗可以起到协同增效作用。PACIFIC研究共纳入了713例局部晚期不可切除的NSCLC患者，结果显示同步放化疗后Durvalumab巩固治疗组的中位PFS分别为23.2个月和17.2个月，中位OS较安慰剂组也显著改善（NR *vs* 29.1个月）^[90]^。PEMBRO-RT研究则是探索了免疫治疗与放化疗同步进行的疗效，晚期肺癌患者在接受立体定位放疗7 d后加用Pembrolizumab，对照组则是仅使用免疫单药，两组的ORR分别为36%和18%，12周DCR分别是64%和40%，中位PFS分别为6.6个月和1.9个月，中位OS分别为15.9个月和7.6个月^[91]^。在晚期肺癌患者中的探索，如免疫代替化疗联合放疗（如NRG LU004 ARCHON-1研究）等均在进行中，期待未来的更多突破。此外，2018年的一项回顾性研究共纳入了26例获得性免疫耐药的NSCLC患者，15例患者在耐药未立即采用全身治疗而选择局部治疗，其中11例患者在局部治疗后继续接受免疫治疗，2年生存率为92%，中位OS尚未达到^[92]^。2020年ASCO会议上的一项回顾性研究得出了相同的结论。该研究共纳入了189例获得性耐药的患者，局部治疗可显著提高生存获益^[93]^，免疫耐药后局部治疗继续联合免疫治疗可在一定程度上逆转耐药，为免疫耐药患者提供了新的治疗思路。

除上述外，其他的联合治疗模式也在对免疫耐药人群进行摸索。如VARGADO研究纳入了57例二线免疫治疗进展后的晚期NSCLC患者，使用VEGF抑制剂尼达尼布联合多西他赛治疗，结果显示中位PFS为6.5个月，中位OS为12.4个月，ORR为50%，DCR为85%^[94]^。尽管目前免疫治疗耐药的研究思路层出不穷，但是逆转免疫耐药的临床试验在开展的同时也有一些不容忽视的问题。①应在试验设计的时候进行充分的临床前评估并在试验进行过程中注意监测试验不良反应。如PACIFIC研究中免疫治疗组放射性肺炎发生率增高（33.9% *vs* 24.8%），有15.4%患者因不良事件中断治疗。TATTON研究中，Durvalumab联合泰瑞沙治疗T790M的NSCLC患者的间质性肺炎的发生率明显增高，甚至高达38%，因此相应的Ⅲ期临床试验（CAURAL研究）中该治疗组被及时中断。此外，克唑替尼联合免疫的治疗，出现了严重的肝损伤。新的治疗策略如免疫联合靶向，需要进一步考虑到不同药物叠加导致的潜在安全性风险。重启免疫治疗前，医生也要考虑到患者的一般情况、全身脏器功能、既往免疫治疗时相关不良反应等情况，再决定治疗方案。②应评估联合策略的合理性，所选的联合治疗策略与免疫检查点抑制剂在免疫环路中是否具有协同增效的作用。例如，增强抗肿瘤细胞抗原性或树突细胞活性的治疗方法与CTLA-4抑制剂的联合作用可能优于PD-1/PD-L1抑制剂，而增强T细胞功能、促进其浸润的方法则与PD-1/PD-L1抑制剂联合更佳。

### 3.2 个体化免疫治疗策略

结合肿瘤免疫微环境的构成特点，研究针对肿瘤细胞新抗原产生及递呈、T细胞活化、局部抑制性微环境解除的药物，如溶瘤病毒、肿瘤疫苗、过继性免疫细胞治疗（包括TIL、CAR-T、TCR-T、CAR-NK等）等基于个体化免疫微环境特征的免疫治疗。一项评价NSCLC患者采用肿瘤浸润淋巴细胞联合Nivolumab进行过继细胞移植的有效性和安全性的试验正在开展。NT-001研究是一项拟探索PD-1抑制剂联合疫苗用于晚期或转移性黑色素瘤、吸烟相关NSCLC和膀胱癌疗效的Ib期临床试验，共纳入了82例患者，在转移性NSCLC患者中，ORR达到了45.5%。MDM2（mouse double minute 2 homolog）是p53的一个最重要的抑制因子，当两者结合时，会使p53蛋白降解、抑癌作用减弱。APG-115是第二代MDM2抑制剂，可以阻断MDM2-p53相互作用，从而恢复p53蛋白的转录调控功能，促进细胞凋亡，恢复肿瘤抑制活性。APG-115联合Pembrolizumab治疗转移性黑色素瘤或晚期实体瘤的Ib期临床试验结果证实，两药联用具有良好的耐受性，ORR为16.7%，DCR为55.5%。随着免疫治疗机制研究的不断深入，个体化免疫治疗靶向较为精准，不失为具有良好发展前景的新策略。

### 3.3 不断开发肿瘤免疫治疗新靶点、新药物

除原有治疗手段以外，不断开发肿瘤免疫治疗的新靶点及新药物也是解决免疫耐药现状的方法之一。腺苷是一种免疫抑制代谢物，可通过与免疫细胞上表达的G蛋白耦联腺苷受体A2a结合，抑制免疫细胞的免疫响应能力，造成肿瘤细胞的免疫逃逸。新型腺苷A2a拮抗剂EOS-850单药治疗耐药实体瘤的临床试验纳入了前列腺癌、结直肠癌、头颈癌、膀胱癌、乳腺癌等多个癌种，试验正在进行中，期待进一步的成果。臂板蛋白4D（semaphorin 4D, SEMA4D）在许多人类肿瘤中广泛表达，其表达与人类侵袭性疾病相关。Pepinemab是首创人源化单抗，可靶向阻断SEMA4D，临床前研究^[95]^显示，其与免疫检查点抑制剂联合用药时可增加免疫细胞浸润，增强ICI效能，延缓肿瘤生长。2020年临床免疫肿瘤学专题研讨会上公布的CLASSICAL-Lung Ib期/Ⅱ期临床试验（NCT03268057）中期分析显示，Pepinemab联合抗PD-L1抑制剂Avelumab治疗晚期NSCLC，在29例可评估的免疫治疗失败的患者中，有59%（17/29）的患者在免疫治疗进展后转向Pepinemab联合Avelumab联合治疗后获得了临床受益，终止或逆转了肿瘤进展。IL-2激动剂（NKTR-214）联合Nivolumab一线或二线治疗晚期肿瘤的研究^[96]^中共纳入了38例晚期肿瘤患者，分别是11例晚期恶性黑色素瘤、22例肾癌和5例NSCLC患者，研究中位随访时间18个月，中位治疗时间13.3个月，在可评估的37例患者中，研究者评估的ORR为59.5%（22/37），疾病控制率为83.8%（31/37）且安全性可靠。组蛋白去乙酰化酶抑制剂（histone deacetylase inhibitors, HDACi）可以通过改变细胞内组蛋白乙酰化程度影响染色质空间结构、染色体重塑，从而抑制目的基因转录，对肿瘤发生、耐药起到关键作用。Mocetinostat是Ⅰ型/Ⅳ型的HDAC抑制剂，在NSCLC小鼠模型中，Mocetinostat可以上调PD-L1表达和抗原递呈，同时可以减少瘤内Treg细胞和MDSC数量、增加瘤内CD8^+^ T细胞的浸润。联合使用Mocetinostat和PD-L1抑制剂可以增强免疫治疗疗效^[97]^，为逆转原发性免疫耐药提供了新的思路。

## 4 总结与展望

免疫治疗相对于传统的化疗及靶向治疗有其独特优势，为肿瘤治疗带来新的曙光。随着免疫治疗的不断推广应用，单药治疗耐药已成为不可回避的问题。对于免疫耐药机制日益深入的研究，也为免疫治疗人群的选择及逆转免疫耐药策略等提供了新的思路。根据患者的耐药类型予以针对性策略，才能提高治疗效率、降低治疗成本，给肿瘤患者带来长期切实的获益。与此同时，不可否认的是对于免疫耐药的研究仍有欠缺之处，如何预测免疫耐药，重启免疫治疗的时机等问题尚未得到合适的回答，需要进一步探索。
